# Validity and Responsiveness of Measuring Facial Swelling With 3D Stereophotogrammetry in Patients After Bilateral Sagittal Split Osteotomy—A Prospective Clinimetric Study

**DOI:** 10.1155/ijbi/9957797

**Published:** 2025-02-24

**Authors:** Margje B. Buitenhuis, Reinoud J. Klijn, Antoine J. W. P. Rosenberg, Caroline M. Speksnijder

**Affiliations:** ^1^Department of Oral and Maxillofacial Surgery, University Medical Center Utrecht, Utrecht University, Utrecht, The Netherlands; ^2^Department of Oral and Maxillofacial Surgery, Medisch Spectrum Twente, Enschede, The Netherlands

**Keywords:** bilateral sagittal split osteotomy, responsiveness stereophotogrammetry, swelling, three-dimensional, validity

## Abstract

**Introduction:** This study is aimed at determining the validity and responsiveness of three-dimensional (3D) stereophotogrammetry as a measurement instrument for evaluating soft tissue changes in the head and neck area.

**Method:** Twelve patients received a bilateral sagittal split osteotomy (BSSO). 3D stereophotogrammetry, tape measurements, and a global perceived effect scale were performed within the first, second, and third postoperative weeks and at 3 months postoperatively. Distance measurements, mean and root mean square of the distance map, and volume differences were obtained from 3D stereophotogrammetry. Validity and responsiveness were assessed by correlation coefficients.

**Results:** Significant correlations between distances from 3D stereophotogrammetry and tape measurements varied from 0.583 to 0.988, meaning moderate to very high validity. The highest correlations were found for the total sum of distances (*r* ≥ 0.922). 3D stereophotogrammetry parameters presented weak to high responsiveness, depending on the evaluated head and neck region. None of the parameters for 3D stereophotogrammetry significantly correlated with the global perceived effect scale outcomes for all measurement moments.

**Conclusion:** 3D stereophotogrammetry has high to very high construct validity for the total sum of distances and weak to high responsiveness. 3D stereophotogrammetry seems promising for measuring soft tissue changes after surgery but is not interchangeable with subjective measurements.

## 1. Introduction

In maxillofacial surgery, the most common orthognathic procedure is the bilateral sagittal split osteotomy (BSSO). Postoperative swelling is a common side effect, mostly expected in the lower face and neck area [1]. Accurate and timely assessment of swelling is clinically relevant for optimizing postoperative care and patient comfort, as it allows clinicians to tailor interventions, and is beneficial for research purposes [2, 3]. When postoperative swelling disappears over time, patients can finally observe surgical soft tissue results. Various methods have been proposed for quantifying swelling in the head and neck region [4]. Tape measurements have high applicability in clinical care because they are inexpensive, simple, and noninvasive [5]. In recent years, advancements in imaging technology have paved the way for noncontact methods, such as optical three-dimensional (3D) scanners, including 3D stereophotogrammetry [5]. 3D stereophotogrammetry has emerged as a promising tool that offers reliable measurements of postoperative swelling in patients after orthognathic surgery, with intraclass correlation coefficients (ICCs) > 0.81 for patients after a BSSO and ICCs ranging from 0.87 to 0.99 for patients after a Le Fort I osteotomy [6, 7]. However, the validity of 3D stereophotogrammetry for measuring facial deformities after orthognathic surgery has only been evaluated to a small extent. Measurements of swelling in the midface with 3D stereophotogrammetry volumetric are strongly correlated with tape measurements for patients after surgically assisted rapid maxillary expansion (SARME) with correlations varying from 0.98 to 0.99 [5]. Nonetheless, the validity of 3D stereophotogrammetry in perimandibular and neck areas has not yet been determined. Besides, changes in swelling over time captured by 3D stereophotogrammetry (responsiveness) and their correlation with patient experience have not yet been evaluated in any applications within the head and neck area. It is important to know the quality of 3D stereophotogrammetry for selecting the best measurement instrument in clinical practice. Therefore, the goal of this study is to determine the construct validity and responsiveness of 3D stereophotogrammetry as a measurement instrument for evaluating soft tissue changes in the head and neck area in patients receiving BSSO advancement.

## 2. Materials and Method

### 2.1. Study Design

This prospective clinimetric study took place at the University Medical Center Utrecht (UMCU), the Netherlands, and utilized a study group similar to that of our previous study [6]. Approval for this study was retrieved from the medical ethical committee of the UMCU (METC Protocol Number 20-780). Written informed consent was obtained from each patient who participated in the study.

The participants comprising 12 consecutive patients receiving a BSSO advancement were included between December 2020 and March 2021. Patients who participated had to be aged at least 18 years and had to be able to sit upright to complete the measurements. Patients were excluded if they had severe facial deformities, substantial facial hair, previous maxillofacial surgery within the 6 months before surgery, or a maxillary osteotomy in addition to the BSSO.

Patients received a BSSO according to the method by Obwegeser with Hunsuck modification [8]. The mandible was placed in the planned position with a 3D-printed surgical splint. Screw fixation was performed with two to four screws per side. Guiding elastics were placed on the orthodontic braces of patients between the maxilla and mandible, and placement was optimized during weekly follow-up. This fixation was phased out after 3 weeks postoperatively. Patients received 12 mg of dexamethasone 15 min before the incision and 8 mg on the first and second postoperative days. Besides, patients were instructed to take 600 mg of ibuprofen three times a day and 1000 mg of paracetamol four times a day during the first postoperative week. Both surgery and follow-up were performed by one experienced oral and maxillofacial surgeon.

### 2.2. Measurements

Validity is defined as the degree to which an instrument measures the construct it aims to measure [9]. Since no gold standard has been established for assessing oedema in the head and neck region [10], the construct validity of 3D stereophotogrammetry was determined by correlating 3D photographs with tape measurements [6, 11]. An aspect of validity is responsiveness, which is defined as the ability of an instrument to detect changes over time in the construct to be measured [12]. The responsiveness of 3D stereophotogrammetry for assessing soft tissue changes was evaluated by correlating measurements with tape measurements and the perception of the patient. The patient's perception was considered an estimate of clinically important change. So, patients were assessed by 3D stereophotogrammetry, tape measurements, and a global perceived effect scale. Measurements were performed within the first (M1), second (M2), and third (M3) postoperative weeks and at 3 months postoperatively (M4) during regular clinical follow-up appointments by one researcher (M.B.B.). A reduction in swelling was expected during the measurements, with the expectation that 80% of the swelling was cleared at M4 [3, 13].

### 2.3. 3D Stereophotogrammetry

3D photographs of the head and neck were captured using the 3dMDface stereophotogrammetry system (3dMD LCC, Atlanta, Georgia, United States) by one experienced observer (M.B.B.) [6]. The 3dMD system consisted of two pods, each equipped with one color and two infrared cameras. This system provided ~190° full face coverage, capturing data from ear to ear. Before use, the camera was calibrated to define a 3D coordinate system.

The 3D photographs were taken with the patient sitting, with hips flexed at 90°, the spine kept vertical, and hands resting on the lap. Seating heights were adjusted to include as much of the neck area as possible. For a standardized reproducible head and upper body orientation, the natural head position was used [14]. The midline of the face was aligned toward the camera, and the patients were asked to look straight ahead at a point at eye level on the wall in front of them. To pursue a reproducible position of the jaws, patients were instructed to slowly close their mouths until first dental contact and then swallow once. The jaws and facial soft tissues were in a relaxed position while gently occluded. Assessment of the 3D photographs was performed directly after capturing to identify and retake 3D photographs with large holes, open mouth, or closed eyes.

### 2.4. 3D Stereophotogrammetry Data Processing

To quantify swelling on 3D photographs, (1) distances were measured on 3D photographs, (2) distance maps were made between 3D photographs, and (3) volume differences were calculated between 3D photographs [6]. Good to excellent reliability has been reported for these outcome parameters with ICCs > 0.81 for distance measurements, ICCs > 0.89 for distance map parameters, and ICCs > 0.90 for volume differences [6]. Outcomes were obtained by processing 3D photographs in 3DMedX (Version 1.2.18.0, Radboudumc, Nijmegen, The Netherlands, https://www.3DMedX.nl, accessed August 18, 2023) and Materialise 3-Matic (Version 15.0, Materialise NV, Leuven, Belgium).

**Figure 1:**
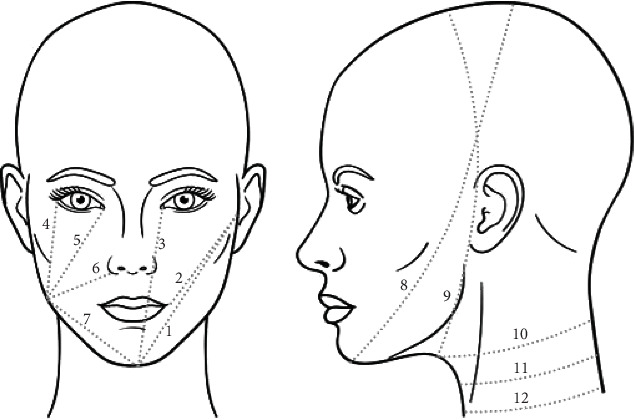
Tape measurements according to the head neck lymphoedema program at MD Anderson Cancer Center with (1) tragus to mental protuberance, (2) tragus to mouth angle, (3) mental protuberance to internal eye corner, (4) mandibular angle to external eye corner, (5) mandibular angle to internal eye corner, (6) mandibular angle to nasal wing, (7) mandibular angle to mental protuberance, (8) diagonal head circumference—chin to crown of the head, (9) vertical head circumference—in front of the ear, (10) superior neck circumference, (11) middle neck circumference, and (12) inferior neck circumference.

Distances on the 3D photographs were retrieved by calculating the shortest distance over the surface between landmarks, following the same landmarks used for the tape measurements [11]. The tape measurements involved seven facial distances (for each facial side), three neck circumferences, and two head circumferences, as depicted in Figure 1. Facial distances were fully performed, while head and neck circumferences were partially measured since only ear-to-ear data was captured.

To obtain distance map parameters and volume differences between two 3D photographs, these photographs were first registered and then split into four regions: right face, left face, submental, and neck. The signed Euclidean distance was calculated from the vertices of each region in a 3D photograph to the closest vertices of the same region in the reference 3D photograph. Visualization of these findings resulted in a distance map. The mean and root mean square (RMS) of the distance map of each region were extracted for data analysis. Volume differences between two 3D photographs were obtained per region by first extruding each region to the same vertical plane and then subtracting the volumes.

For the comparison with tape measurements, distance map parameters and volume differences were calculated from 3D photographs at M1, M2, and M3 to the reference photograph at M4. For the comparison with the global perceived effect scale, the difference in distance measurements, distance map parameters, and volume differences were calculated from 3D photographs at M2, M3, and M4 to the photograph at M1 since the effect scale was also scored related to the first measurement moment.

### 2.5. Tape Measurements

Tape measurements were performed following the head and neck lymphoedema program at MD Anderson Cancer Center, using a soft vinyl medical measuring tape [11]. These measurements have good to excellent reliability for neck circumferences (ICCs ≥ 0.90), good to moderate reliability for head circumferences (ICCs 0.70–0.81), and poor to moderate reliability for facial distances (ICCs 0.33–0.70) [15]. Tape measurements were performed with the patient positioned in the same manner as during the 3D stereophotogrammetry measurements. Each distance was measured twice per measurement moment of which the mean was used for data analysis.

### 2.6. Global Perceived Effect Scale on Swelling Change

Patients scored their perception of change in swelling based on the 7-point global perceived effect scale [16, 17]. The scale was divided into (1) more swollen than ever, (2) much more swollen, (3) slightly more swollen, (4) unchanged, (5) slightly less swollen, (6) much less swollen, and (7) completely recovered. Patients were asked to compare their current swelling with that at the first postoperative measurement (M1).

### 2.7. Statistical Analysis

All analyses were conducted using Statistical Package for the Social Sciences (SPSS) (Version 27, IBM, Chicago, Illinois, United States). *p* values below 0.05 were considered statistically significant. A power analysis was performed with a significance level of 5%, power of 80%, and an expected correlation of at least 0.72. Previously reported correlations between 3D stereophotogrammetry and tape measurements in the midface were at least 0.98; however, since this is based on a single study, we conservatively anticipated a correlation of at least 0.72 [5]. The required sample size was determined to be a minimum of 12 participants.

Baseline characteristics were presented by mean and standard deviation (SD) for normally distributed continuous data and median and interquartile range (IQR) for nonnormally distributed continuous data. The mean and SD or median and IQR of the distances, mean distance map, RMS distance map, and volume differences were calculated for each measurement moment. Significant differences between measurement moments were analyzed using repeated measures analysis of variance (ANOVA).

For the evaluation of the construct validity and responsiveness, the Pearson correlation coefficient (*r*_p_) was obtained for normally distributed continuous data and the Spearman correlation coefficient (*r*_s_) for ordinal and nonnormally distributed continuous data [18]. The normal distribution of parameters was assessed using the Shapiro–Wilk test.

To test construct validity, the distance measurements of the 3D stereophotogrammetry and tape measurements were correlated per measurement moment. Besides, correlations were determined for the sum of distances per distance category (right face, left face, head circumference, and neck circumference) and the total sum of all distance measurements.

For testing responsiveness, the distance map parameters and volume differences (right face, left face, submental, and neck) between two 3D photographs were correlated with the distance differences obtained from tape measurements. Furthermore, distance map parameters, volume differences, and differences in distance measurements from 3D stereophotogrammetry were correlated with the global perceived effect scale. For the distance map parameters, correlations were determined per region, while for volume difference, correlations were determined per region and for the sum of all regions. The difference in distance measurements was correlated for each measurement, the sum of distances per distance category, and the total sum of all distance measurements. In addition, tape measurements were correlated with the global perceived effect scale.

For significant correlations, the strength of the correlation was interpreted as weak (< 0.35), moderate (0.36–0.67), high (0.68–0.89), and very high (≥ 0.90) based on the absolute value of the correlation coefficient [19].

## 3. Results

**Figure 2:**
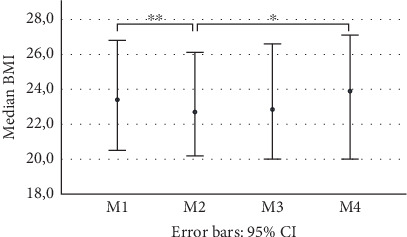
Body mass index (BMI) of included patients (*n* = 12) at measurement moments during the first (M1), second (M2), and third (M3) postoperative weeks and 3 months postoperative (M4). ⁣^∗^Significant with *p* < 0.05; ⁣^∗∗^significant with *p* < 0.01.

Twelve patients were included in the data analysis, comprising five males and seven females. All patients were scheduled for a BSSO, with a median advancement of 6 mm (range 2.5–9.0 mm). The median age was 27 years, with an IQR of 24–48. The mean body mass index (BMI) was 23.9 (SD 4.2) at M1, 23.5 (SD 4.3) at M2, 23.5 (SD 4.4) at M3, and 24.1 (SD 4.4) at M4. BMI significantly differed between M1 and M2 (*p* = 0.008) and between M2 and M4 (*p* = 0.041) (Figure 2). No patients were excluded during the study.

In Table 1, the mean and SD or median and IQR of the distances obtained from 3D stereophotogrammetry and tape measurements are demonstrated. For both 3D stereophotogrammetry and tape measurements, 14 out of the 19 distances significantly varied between measurements. Besides, the sum of distances per category significantly differed between the measurements (*p* values ≤ 0.011), with the distances decreasing over time.

Table 2 presents the mean and RMS distance map and volume difference for each region, which reduced over time, with significant differences between measurements (*p* values < 0.001).

### 3.1. Construct Validity

See Table 3 for correlations between distances obtained from 3D stereophotogrammetry and tape measurements. The correlations were very high for the total sum of all distances and high to very high for the sum of distances per category. All correlations were statistically significant, except for the right face measurement of the “mandibular angle to nasal wing” (tape measurement 6) and “mandibular angle to mental protuberance” (tape measurement 7) at M1 and the “inferior neck circumference” at M1 and M3. Significant correlations ranged from 0.619 to 0.967 for right facial measurements, from 0.583 to 0.988 for left facial measurements, from 0.625 to 0.965 for neck circumferences, and from 0.652 to 0.876 for head circumferences.

### 3.2. Responsiveness

In Table 4, the mean and RMS of the distance map and volume differences from 3D stereophotogrammetry are presented. Significant correlations with the sum of distances from tape measurements were only found for the submental region at M1 (mean of distance map: *r*_p_ = 0.755; RMS of distance map: *r*_p_ = 0.755) and at M2 (mean of distance map: *r*_p_ = 0.701; volume difference: *r*_p_ = 0.658). Distance map parameters and volume differences from 3D stereophotogrammetry presented significant moderate to high correlations with nine differences in distances from tape measurements at M1, five at M2, and two at M3. All other correlations with individual distance tape measurements were not significant.

**Figure 3:**
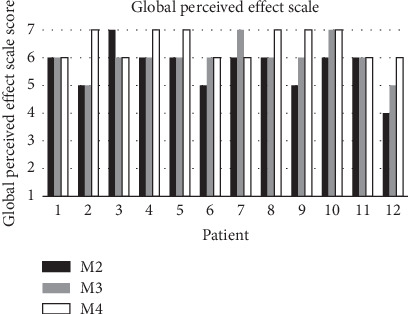
Global perceived effect scale scores at the second (M2) and third (M3) postoperative weeks and 3 months postoperative (M4) related to the first postoperative week (M1).

Compared to the first postoperative measurement, one patient scored swelling as unchanged, three patients as slightly less swollen, seven patients as much less swollen, and one patient as completely recovered at M2, as depicted in Figure 3. At M3, two patients scored their swelling as slightly less swollen, eight patients as much less swollen, and two patients as completely recovered. At M4, half of the patients scored their swelling as much less swollen and half as completely recovered. When correlating 3D stereophotogrammetry with this perceived global effect scale, none of the parameters correlated significantly at all measurement moments.

Table 5 displays the correlations between difference in the distance measurements from 3D stereophotogrammetry and the perceived global effect scale. Significant correlations were observed for only the left measurement of the “mandibular angle to external eye corner” (tape measurement 4) at M2 (*r*_s_ = −0.661) and M3 (*r*_s_ = −0.669) and the left “mandibular angle to nasal wing” (tape measurement 6) at M3 (*r*_s_ = −0.711). The mean distance map significantly correlated with the perceived effect scale for only the submental region at M1 (*r*_s_ = −0.629) and the neck region at M2 (*r*_s_ = −0.669). Significant correlations were found for the RMS distance map for only the left face region at M1 (*r*_s_ = 0.595) and for volume differences only for the neck region at M2 (*r*_s_ = −0.627). All other correlations were not significant.

Table 6 presents the correlations between tape measurements and the perceived global effect scale, demonstrating only significant correlations for the sum of distances of the right face (*r*_s_ = −0.615) and left face (*r*_s_ = −0.646) at M2.

## 4. Discussion

This study is aimed at assessing the validity and responsiveness of 3D stereophotogrammetry in patients receiving a BSSO advancement. The most important finding of this study is the high to very high construct validity of 3D stereophotogrammetry for measuring swelling in the head and neck area for the total sum of distances. The mean and RMS of the distance map and volume differences presented weak to high responsiveness, depending on the evaluated region. The patient's perception did not significantly correlate with any of the 3D stereophotogrammetry parameters or tape measurements for all measurement moments.

### 4.1. Construct Validity

Very high correlations between 3D stereophotogrammetry and tape measurements were found for the total sum of distances, presenting a very high construct validity. These correlations varied only slightly between measurements at the different measurement moments, suggesting that the construct validity was only minimally affected by the presence of postoperative swelling. Construct validity for the distances per category (right face, left face, head circumference, and neck circumference) varied more over time, with increasing quality of the construct validity during the postoperative course. Besides, the absolute values of all 3D stereophotogrammetry outcome parameters decreased over a 3-month period. These varying levels of construct validity and 3D stereophotogrammetry outcomes imply that the presence of postoperative swelling negatively affected the correlations between these distances obtained from 3D stereophotogrammetry and tape measurements. A larger amount of soft tissue swelling could hamper the palpation of the skeletal landmarks during tape measurements and impair the visibility of these landmarks at 3D photographs. The correlations for individual facial distance measurements were weakest for the distances from “mandibular angle to nasal wing” (tape measurement 6) and “mandibular angle to mental protuberance” (tape measurement 7) at the first postoperative measurement. This lower correlation may be a consequence of a higher measurement error [20]. Earlier research demonstrated that the measurement error for tape measurements was largest for the distance from gonion to pogonion (corresponding with tape measurement 7) for all facial distances [15]. In addition, research on the reliability of 3D stereophotogrammetry measurements demonstrated that tape measurements 6 and 7 presented the lowest, but still good, reliability (ICCs > 0.81) [6]. The largest measurement error and lowest reliability for these tape measurements correspond to the weakest construct validity found in this study. However, the effect of lower correlations at measurement moments when more postoperative swelling was expected was not observed for the head and neck circumference measurements.

The sum of circumferences presented somewhat lower construct validity compared to the sum of facial distances. This difference may be attributed to altered measurements at the 3D photographs since the 3dMDface stereophotogrammetry system only included ear-to-ear data. Moreover, the height of the middle and inferior neck circumferences at 3D photography probably differed from the height during tape measurements because the entire neck could not be captured with the 3dMDface stereophotogrammetry system.

To our knowledge, this is the first study correlating distances from 3D stereophotogrammetry with distances from tape measurements in patients. For healthy subjects, there is one study that correlated other distance measurements in the midface from 3D stereophotogrammetry with tape measurements, resulting *r*_p_ values between 0.49 and 0.98, which is a somewhat narrower range than what was found in our study [21]. This difference may be explained by variations in the measurements being evaluated.

### 4.2. Responsiveness

Distance map parameters and volume differences presented significant moderate to high correlations for the submental area with the “vertical head circumference” from tape measurements at M1. Some of the 3D stereophotogrammetry parameters were significant at other measurement moments, but none of the parameters correlated significantly with any of the distances from tape measurements for all measurement moments. This could be the consequence of disparities in considered regions. Distance map and volumetric parameters encompassed the whole facial, submental, or neck region, while tape measurements only covered specific local distances. The head circumference tape measurement fits best with the analysis of the submental region.

It was expected that the correlations between 3D stereophotogrammetry measurements and the perceived global effect scale would be better for distance map parameters and volume differences compared to distance measurements. This was because the face is rather processed as a whole than as a combination of parts [22]. However, none of the parameters from 3D stereophotogrammetry significantly correlated with the patient's perception for all measurement moments. This may be due to small spread in the patient's perception.

The lack of correlation between the patient's perception and both 3D stereophotogrammetry and tape measurements suggests that these are not interchangeable. This finding aligns with previous research that demonstrated poor correlations between objective and subjective measures, underpinning the importance of combining clinical measures and patient-reported outcomes [23–25].

### 4.3. Strengths and Limitations

This study evaluated an increasingly utilized technique for a new application. The research is closely connected to clinical practice by comparing it with the highest clinically applicable measurement available. The method used for data analysis is extensively reported and thereby reproducible [6]. Nevertheless, this study also had some limitations. Postoperative swelling was not the sole varying parameter during the postoperative course in patients, so outcomes may have been affected by other factors. BMI significantly differed between measurements, potentially attributed to the required dietary changes, restricting the consumption of hard foods postoperatively. Patients who undergo orthognathic surgery with a higher BMI tend to experience more postoperative swelling and a faster reduction of swelling [3]. Furthermore, the impact of age, dietary and fluid intake, menstrual cycle, and activity level was not investigated in this study [26–28]. Examining the influence of these effects on the measurement properties of 3D stereophotogrammetry was not feasible due to the relatively small sample size. 3D stereophotogrammetry, as a measurement instrument for soft tissue swelling, had certain restrictions. The 3dMDface stereophotogrammetry system could only capture ear-to-ear data. Moreover, some areas could not be captured in all individuals, for example, the submental and submandibular areas in thin patients with well-defined jawlines. In patients with more soft tissue, attributed to a higher BMI or swelling, the identification of skeletal landmarks at 3D photographs was experienced more difficult since bone structures were less visible.

### 4.4. Recommendations

This study revealed the validity and responsiveness of 3D stereophotogrammetry in quantifying soft tissue swelling in patients receiving a BSSO advancement. Before the clinical application of 3D stereophotogrammetry for quantifying soft tissue swelling in patients after BSSO, the minimal important change (MIC) should be determined. MIC is the smallest change in soft tissue swelling to be measured which patients perceive as important. When the MIC exceeds the previously reported smallest detectable change (SDC) [6], 3D stereophotogrammetry adds value in measuring soft tissue swelling.

To detect changes in soft tissue swelling with 3D stereophotogrammetry, measurements should be performed over time. For accurate interpretation, it should be possible to distinguish between changes in soft tissue and changes resulting from variations in head and neck positions. Therefore, it is recommended to quantify positions after applying the natural head position and standardizing the neck position. A 3D stereophotogrammetry system that captures 360° of data could simplify interpretation.

### 4.5. Clinical Implications

3D stereophotogrammetry might be more comfortable for the patient compared to tape measurements since it is a noncontact method and measurements take less time to perform. Nonetheless, the analysis of 3D stereophotogrammetry also takes time, which could hamper applicability in primary care. However, the analysis is semiautomatic and could be further automated with the rise of artificial intelligence in the medical sector. 3D stereophotogrammetry could also be explored for applications in patients with head and neck cancer to quantify lymphoedema. It could be beneficial to decide on interventions for swelling reduction based on the combination of objective and subjective measurements of swelling.

## 5. Conclusions

3D stereophotogrammetry is promising for measuring soft tissue changes caused by swelling in the lower face and neck areas after orthognathic surgery. This study demonstrated that 3D stereophotogrammetry has high to very high construct validity for the total sum of distances. The ability of 3D stereophotogrammetry to measure changes over time was weak to high, depending on the evaluated region of the head and neck. The global perceived effect scale did not significantly correlate with any of the 3D stereophotogrammetry parameters or tape measurements for all measurement moments, suggesting that objective and subjective measurements are not interchangeable.

## Figures and Tables

**Table 1:** Distances from 3D stereophotogrammetry and tape measurements.

**Distance measurement**		**Distances (mm)**									
		**3D stereophotogrammetry**					**Tape measurements**				
		**M1**	**M2**	**M3**	**M4**	**p** ** value**	**M1**	**M2**	**M3**	**M4**	**p** ** value**
Right face	1	154 ± 8.0	153 ± 7.1	152 ± 7.9	152 ± 8.6	0.058	157 ± 8.9	155 ± 8.0	154 ± 9.0	153 ± 9.0	0.001⁣^∗∗^
	2	118 ± 7.6	117 ± 6.6	117 ± 7.5	116 ± 8.3	0.047⁣^∗^	118 ± 6.9	116 ± 6.8	115 ± 7.0	114 ± 7.8	0.006⁣^∗∗^
	3	115 ± 7.8	115 ± 8.5	113 ± 8.7	113 ± 9.1	0.202	111 ± 8.0	110 ± 8.3	111 ± 7.9	110 ± 8.0	0.732
	4	112 ± 6.5	108 (106–111)^a^	109 ± 6.3	108 ± 7.5	0.017⁣^∗^	101 ± 5.9	98 ± 5.6	96 ± 6.0	97 ± 5.3	0.001⁣^∗∗^
	5	138 ± 7.4	134 ± 7.2	134 ± 6.4	133 ± 7.0	0.001⁣^∗∗^	127 ± 6.3	127 ± 6.2	125 ± 6.1	124 ± 6.2	0.049⁣^∗^
	6	119 ± 5.1	117 ± 5.1	116 ± 4.6	115 ± 4.7	0.000⁣^∗∗∗^	112 ± 6.3	112 ± 5.1	109 ± 5.9	109 ± 7.2	0.013⁣^∗^
	7	118 ± 8.0	116 ± 8.4	114 ± 6.7	115 ± 7.4	0.083	122 (116–125)^a^	118 ± 7.5	115 ± 7.9	110 ± 7.2	0.000⁣^∗∗∗^
	Sum	873 ± 42	861 ± 44	856 ± 41	851 ± 46	0.000⁣^∗∗∗^	845 ± 40	835 ± 39	825 ± 42	817 ± 41	0.000⁣^∗∗∗^

Left face	1	154 ± 7.3	154 ± 8.5	153 ± 8.7	152 ± 8.5	0.002⁣^∗∗^	156 ± 7.5	154 ± 9.5	153 ± 9.1	152 ± 9.1	0.000⁣^∗∗∗^
	2	119 ± 7.0	118 ± 6.9	117 ± 8.2	116 ± 8.0	0.015⁣^∗^	119 ± 5.8	117 ± 6.7	117 ± 5.9	114 ± 7.5	0.000⁣^∗∗∗^
	3	112 ± 8.1	113 ± 8.4	113 ± 8.7	112 ± 9.1	0.568	109 ± 7.3	109 ± 8.1	110 ± 9.0	109 ± 8.8	0.776
	4	110 ± 4.9	108 ± 7.5	108 ± 5.3	106 ± 5.9	0.011⁣^∗^	100 ± 4.3	95 (93–102)^a^	95 ± 5.2	95 ± 6.9	0.016⁣^∗^
	5	137 ± 6.1	134 ± 7.2	133 ± 5.7	131 ± 6.9	0.001⁣^∗∗^	125 ± 6.8	124 ± 6.6	124 ± 5.7	123 ± 7.7	0.315
	6	120 ± 6.4	117 ± 5.9	115 ± 5.0	113 ± 6.9	0.000⁣^∗∗∗^	109 ± 7.3	113 (105–114)^a^	108 ± 6.6	106 ± 7.3	0.045⁣^∗^
	7	120 ± 8.5	117 ± 9.4	117 ± 7.4	115 ± 8.3	0.001⁣^∗∗^	114 ± 7.9	112 ± 5.9	113 ± 7.8	104 ± 6.9	0.000⁣^∗∗∗^
	Sum	874 ± 41	861 ± 48	855 ± 42	845 ± 48	0.000⁣^∗∗∗^	831 ± 37	824 ± 42	819 ± 41	802 ± 47	0.000⁣^∗∗∗^

Neck circumference	Superior	227 ± 26	215 ± 25	221 (189–230)^a^	206 ± 26	0.000⁣^∗∗∗^	365 ± 41	359 ± 39	359 ± 42	357 ± 40	0.005⁣^∗∗^
	Middle	195 ± 27	188 ± 23	187 ± 24	180 ± 24	0.006⁣^∗∗^	359 ± 41	346 ± 56	344 ± 57	352 ± 38	0.130
	Inferior	199 ± 30	192 ± 23	193 ± 28	182 ± 25	0.035⁣^∗^	365 ± 36	358 ± 34	357 ± 36	359 ± 36	0.014⁣^∗^
	Sum	621 ± 78	595 ± 67	592 ± 71	568 ± 71	0.000⁣^∗∗∗^	1089 ± 117	1063 ± 125	1059 ± 130	1068 ± 113	0.011⁣^∗^

Head circumference	Diagonal	339 ± 19	338 ± 19	342 ± 29	338 ± 21	0.430	678 (625–692)^a^	659 ± 34	656 ± 36	657 (621–686)^a^	0.417
	Vertical	354 ± 29	338 ± 26	337 ± 25	331 ± 29	0.000⁣^∗∗∗^	665 ± 38	644 ± 37	643 ± 36	635 ± 29	0.000⁣^∗∗∗^
	Sum	692 ± 48	675 ± 44	679 ± 53	669 ± 48	0.000⁣^∗∗∗^	1324 ± 75	1310 (1224–1366)^a^	1299 ± 67	1290 ± 58	0.000⁣^∗∗∗^

*Note:* Data are retrieved at measurement moments M1–M3 (first–third postoperative week) and M4 (3 months postoperative). Repeated measures ANOVA was used to analyze differences over time. Data are presented with the mean ± standard deviation (no alphanumerical superscript) or median (interquartile range) (superscript a).

⁣^∗^Significant with *p* < 0.05.

⁣^∗∗^Significant with *p* < 0.01.

⁣^∗∗∗^Significant with *p* < 0.001.

**Table 2:** Mean distance map, root mean square distance map, and volume from 3D stereophotogrammetry per region.

**Parameter**	**Region**	**M1**	**M2**	**M3**	**p** ** value**
Mean distance map (mm)	Right face	1.27 ± 0.85	0.66 ± 0.56	0.38 ± 0.36	0.000⁣^∗∗∗^
	Left face	1.53 ± 0.77	0.82 ± 0.49	0.63 ± 0.33	0.000⁣^∗∗∗^
	Submental	5.53 ± 1.9	2.74 ± 0.80	1.94 ± 0.80	0.000⁣^∗∗∗^
	Neck	7.16 ± 4.7	4.40 ± 3.0	3.17 ± 3.5	0.000⁣^∗∗∗^

Root mean square distance map	Right face	2.24 ± 0.73	1.65 ± 0.51	1.05 ± 0.39	0.000⁣^∗∗∗^
	Left face	2.60 ± 0.79	1.73 ± 0.81	1.40 ± 0.50	0.000⁣^∗∗∗^
	Submental	6.32 ± 1.8	3.49 ± 0.98	3.27 ± 1.8	0.000⁣^∗∗∗^
	Neck	11.3 ± 4.5	8.84 ± 3.6	7.66 ± 3.8	0.000⁣^∗∗∗^

Volume difference (mL)	Right face	13.0 ± 10	5.4 ± 6.6	2.9 ± 4.7	0.000⁣^∗∗∗^
	Left face	16.7 ± 7.9	8.8 ± 5.5	7.1 ± 4.0	0.000⁣^∗∗∗^
	Submental	39.0 ± 18	16.2 (12.6–25.4)^a^	12.2 ± 5.8	0.000⁣^∗∗∗^
	Neck	82.5 ± 55	43.3 ± 35	30.2 ± 44	0.000⁣^∗∗∗^

*Note:* Data present the difference from measurement moments M1–M3 (first–third postoperative week) to the reference at M4 (3 months postoperative). Repeated measures ANOVA was used to analyze differences over time. Data are presented with the mean ± standard deviation (no alphanumerical superscript) or median (interquartile range) (superscript a).

⁣^∗∗∗^Significant with *p* < 0.001.

**Table 3:** Correlations between distances from 3D stereophotogrammetry and tape measurements.

**Distance measurement**		**Correlation coefficient**			
		**M1**	**M2**	**M3**	**M4**
Right face	1	0.899⁣^∗∗∗^	0.927⁣^∗∗∗^	0.965⁣^∗∗∗^	0.908⁣^∗∗∗^
	2	0.967⁣^∗∗∗^	0.937⁣^∗∗∗^	0.931⁣^∗∗∗^	0.933⁣^∗∗∗^
	3	0.898⁣^∗∗∗^	0.869⁣^∗∗∗^	0.959⁣^∗∗∗^	0.932⁣^∗∗∗^
	4	0.833⁣^∗∗^	0.674^a^⁣^∗^	0.860⁣^∗∗∗^	0.833⁣^∗∗^
	5	0.644⁣^∗^	0.863⁣^∗∗∗^	0.879⁣^∗∗∗^	0.791⁣^∗∗^
	6	0.241	0.682⁣^∗^	0.814⁣^∗∗^	0.776⁣^∗∗^
	7	0.560^a^	0.619⁣^∗^	0.857⁣^∗∗∗^	0.797⁣^∗∗^
	Sum	0.914⁣^∗∗∗^	0.910⁣^∗∗∗^	0.969⁣^∗∗∗^	0.937⁣^∗∗∗^

Left face	1	0.969⁣^∗∗∗^	0.988⁣^∗∗∗^	0.966⁣^∗∗∗^	0.978⁣^∗∗∗^
	2	0.925⁣^∗∗∗^	0.957⁣^∗∗∗^	0.929⁣^∗∗∗^	0.956⁣^∗∗∗^
	3	0.909⁣^∗∗∗^	0.890⁣^∗∗∗^	0.947⁣^∗∗∗^	0.934⁣^∗∗∗^
	4	0.612⁣^∗^	0.872^a^⁣^∗∗∗^	0.706⁣^∗^	0.881⁣^∗∗∗^
	5	0.661⁣^∗^	0.803⁣^∗∗^	0.843⁣^∗∗^	0.867⁣^∗∗∗^
	6	0.756⁣^∗∗^	0.592^a^⁣^∗^	0.623⁣^∗^	0.753⁣^∗∗^
	7	0.583⁣^∗^	0.613⁣^∗^	0.842⁣^∗∗^	0.883⁣^∗∗∗^
	Sum	0.917⁣^∗∗∗^	0.926⁣^∗∗∗^	0.959⁣^∗∗∗^	0.969⁣^∗∗∗^

Neck circumference	Superior	0.870⁣^∗∗∗^	0.935⁣^∗∗∗^	0.846^a^⁣^∗∗^	0.965⁣^∗∗∗^
	Middle	0.821⁣^∗∗^	0.726⁣^∗∗^	0.751⁣^∗∗^	0.885⁣^∗∗∗^
	Inferior	0.514	0.625⁣^∗^	0.508	0.711⁣^∗^
	Sum	0.768⁣^∗∗^	0.834⁣^∗∗^	0.797⁣^∗∗^	0.903⁣^∗∗∗^

Head circumference	Diagonal	0.748^a^⁣^∗∗^	0.876⁣^∗∗∗^	0.773⁣^∗∗^	0.834^a^⁣^∗∗^
	Vertical	0.815⁣^∗∗^	0.652⁣^∗^	0.663⁣^∗^	0.821⁣^∗∗^
	Sum	0.832⁣^∗∗^	0.788^a^⁣^∗∗^	0.791⁣^∗∗^	0.891⁣^∗∗∗^

Total sum		0.939⁣^∗∗∗^	0.922⁣^∗∗∗^	0.943⁣^∗∗∗^	0.970⁣^∗∗∗^

*Note:* Data are retrieved at measurement moments M1–M3 (first–third postoperative week) and M4 (3 months postoperative). Correlations are presented with the Pearson correlation coefficient (no alphanumerical superscript) or Spearman correlation coefficient (superscript a).

⁣^∗^Significant with *p* < 0.05.

⁣^∗∗^Significant with *p* < 0.01.

⁣^∗∗∗^Significant with *p* < 0.001.

**Table 4:** Correlations between distance map and volumetric parameters from 3D stereophotogrammetry and distance differences from tape measurements at measurement moments M1–M3 (first–third postoperative week) to the reference at M4 (3 months postoperative).

**3D stereophotogrammetry**	**Tape measurement**		**Correlation coefficient**		
**Parameter**	**Distance category**	**Measurement**	**M1**	**M2**	**M3**
Mean distance map right face	Right face	1	0.534	0.017	0.520
		2	0.621⁣^∗^	0.509	0.575
		3	0.068^a^	0.032	−0.210
		4	0.524	−0.058	−0.017
		5	0.202	−0.118	−0.293
		6	−0.082	−0.042	0.090
		7	0.220	0.336	−0.136^a^
		Sum	0.415	0.157	0.316

Mean distance map left face	Left face	1	0.451	−0.128^a^	0.457
		2	0.879⁣^∗∗∗^	0.602⁣^∗^	0.471
		3	0.330	−0.328	0.422
		4	0.251	−0.023	0.167
		5	0.121	−0.231	−0.084
		6	0.524	0.431	−0.017
		7	0.471	0.308	0.330
		Sum	0.543	0.336^a^	0.437

Mean distance map submental	Head circumference	Diagonal	0.602⁣^∗^	0.300	−0.135
		Vertical	0.718⁣^∗∗^	0.703⁣^∗^	0.545
		Sum	0.755⁣^∗∗^	0.701⁣^∗^	0.353

Mean distance map neck	Neck circumference	Superior	0.311	0.276	0.043
		Middle	0.539	0.266^a^	0.532^a^
		Inferior	0.159	0.405	0.313
		Sum	0.409	0.329	0.558

Root mean square distance map right face	Right face	1	0.456	0.154	0.305
		2	0.412	−0.130	0.309
		3	0.140	0.374	−0.216
		4	0.532	−0.084	−0.023
		5	0.234	−0.033	−0.380
		6	−0.067	0.224	−0.106
		7	0.290	0.300	−0.250
		Sum	0.410	0.575	−0.033

Root mean square distance map left face	Left face	1	0.259	−0.135^a^	0.613⁣^∗^
		2	0.876⁣^∗∗∗^	0.535^a^	0.330
		3	0.385	−0.320^a^	−0.081
		4	0.042	−0.119^a^	0.063
		5	−0.164	−0.585^a^⁣^∗^	−0.294
		6	0.397	0.217^a^	−0.328
		7	0.417	0.396^a^	0.059
		Sum	0.365	0.196^a^	0.025

Root mean square distance map submental	Head circumference	Diagonal	0.610⁣^∗^	−0.120^a^	−0.006
		Vertical	0.712⁣^∗∗^	0.193^a^	0.182
		Sum	0.755⁣^∗∗^	0.060^a^	0.141

Root mean square distance map neck	Neck circumference	Superior	0.275	−0.240	0.000
		Middle	0.493	0.196	0.291
		Inferior	0.309	0.326	0.398
		Sum	0.425	0.190	0.361

Volume difference right face	Right face	1	0.444	0.061	0.557
		2	0.396	0.215	0.360
		3	0.085	−0.167	−0.265
		4	0.362	−0.253	−0.137
		5	0.052	−0.201	−0.353
		6	−0.260	−0.040	0.444
		7	0.335	0.226	0.354
		Sum	0.274	−0.052	0.327

Volume difference left face	Left face	1	0.459	0.366	0.569
		2	0.841⁣^∗^	0.645⁣^∗^	0.364
		3	0.307	−0.361	0.337
		4	0.138	−0.091	0.102
		5	0.020	−0.299	−0.226
		6	0.411	0.325	−0.138
		7	0.396	0.248	0.274
		Sum	0.441	0.106	0.292

Volume difference submental	Head circumference	Diagonal	0.378	0.285^a^	−0.010
		Vertical	0.608⁣^∗^	0.711^a^⁣^∗^	0.651⁣^∗^
		Sum	0.572	0.658^a^⁣^∗^	0.510

Volume difference neck	Neck circumference	Superior	0.263	0.423	−0.077
		Middle	0.498	0.200	0.417
		Inferior	0.121	0.449	0.242
		Sum	0.344	0.384	0.419

*Note:* Correlations are presented with the Pearson correlation coefficient (no alphanumerical superscript) or Spearman correlation coefficient (superscript a).

⁣^∗^Significant with *p* < 0.05.

⁣^∗∗^Significant with *p* < 0.01.

⁣^∗∗∗^Significant with *p* < 0.001.

**Table 5:** Spearman correlations between distance difference, distance map, and volumetric parameters from 3D stereophotogrammetry and the perceived global effect scale.

**Parameter 3D stereophotogrammetry**			**Spearman correlation coefficient**		
			**M2**	**M3**	**M4**
Distances	Right face	1	−0.047	−0.125	0.024
		2	−0.181	0.125	−0.241
		3	−0.260	−0.419	−0.339
		4	−0.252	−0.209	−0.218
		5	−0.134	−0.167	−0.290
		6	−0.024	0.084	−0.169
		7	−0.181	−0.084	−0.193
		Sum	−0.047	−0.209	−0.290
	Left face	1	−0.378	0.125	−0.290
		2	−0.330	−0.167	0.193
		3	−0.024	0.000	−0.441
		4	−0.661⁣^∗^	0.669⁣^∗^	−0.048
		5	−0.283	0.334	−0.048
		6	−0.110	0.711⁣^∗^	−0.048
		7	−0.236	0.502	−0.338
		Sum	−0.417	0.502	−0.097
	Neck circumference	Superior	−0.535	−0.334	−0.048
		Middle	−0.252	−0.460	−0.073
		Inferior	−0.497	−0.125	−0.097
		Sum	−0.496	−0.314	−0.097
	Head circumference	Diagonal	−0.378	0.334	0.000
		Vertical	−0.362	−0.125	−0.145
		Sum	−0.417	0.418	−0.290
	Total sum		−0.548	0.167	−0.193

Mean distance map	Right face		−0.401	−0.167	−0.048
	Left face		−0.536	−0.084	0.097
	Submental		−0.629⁣^∗^	−0.209	−0.531
	Neck		−0.378	−0.669⁣^∗^	−0.145

Root mean square distance map	Right face		−0.236	0.063	0.048
	Left face		0.595⁣^∗^	0.418	−0.145
	Submental		0.260	−0.084	0.531
	Neck		0.055	−0.042	0.386

Volume difference	Right face		−0.291	−0.398	−0.097
	Left face		−0.544	0.021	0.048
	Submental		−0.472	−0.209	−0.483
	Neck		−0.220	−0.627⁣^∗^	0.048
	Sum		−0.448	−0.334	−0.145

*Note:* Data are retrieved between measurement moments M2, M3, and M4 (second and third postoperative weeks and third postoperative month) and M1 (first postoperative week).

⁣^∗^Significant with *p* < 0.05.

**Table 6:** Spearman correlations between tape measurements and the perceived global effect scale.

**Tape measurement**		**Spearman correlation coefficient**		
		**M2**	**M3**	**M4**
Right face	1	0.055	0.444	0.556
	2	−0.203	0.337	−0.049
	3	−0.550	−0.232	−0.025
	4	−0.303	−0.105	−0.073
	5	−0.478	0.021	0.048
	6	−0.330	0.335	0.509
	7	−0.119	−0.189	0.024
	Sum	−0.615⁣^∗^	0.042	0.193

Left face	1	−0.119	0.042	−0.459
	2	−0.533	0.000	0.365
	3	−0.327	−0.021	−0.267
	4	−0.497	0.293	−0.218
	5	−0.219	0.356	−0.145
	6	−0.095	0.105	0.048
	7	−0.067	0.230	−0.145
	Sum	−0.646⁣^∗^	0.334	−0.073

Neck circumference	Superior	−0.087	−0.105	0.411
	Middle	−0.319	−0.021	0.097
	Inferior	−0.304	0.209	−0.193
	Sum	−0.244	0.167	0.145

Head circumference	Diagonal	−0.275	0.209	−0.459
	Vertical	−0.299	−0.377	−0.193
	Sum	−0.299	−0.042	−0.338

Total sum		−0.346	0.251	−0.145

*Note:* Data are retrieved between measurement moments M2, M3, and M4 (second and third postoperative weeks and third postoperative month) and M1 (first postoperative week).

⁣^∗^Significant with *p* < 0.05.

## Data Availability

The 3D stereophotogrammetry data of the head and neck used to support the findings of this study have not been made available because of the privacy of the participants. The other data used to support the findings of this study are available from the corresponding author upon request.

## References

[1] Semper-Hogg W., Fuessinger M. A., Dirlewanger T. W., Cornelius C. P., Metzger M. C. (2017). The influence of dexamethasone on postoperative swelling and neurosensory disturbances after orthognathic surgery: a randomized controlled clinical trial. *Head & Face Medicine*.

[2] Rana M., Gellrich N. C., Joos U., Piffkó J., Kater W. (2011). 3D evaluation of postoperative swelling using two different cooling methods following orthognathic surgery: a randomised observer blind prospective pilot study. *International Journal of Oral and Maxillofacial Surgery*.

[3] Van Der Vlis M., Dentino K. M., Vervloet B., Padwa B. L. (2014). Postoperative swelling after orthognathic surgery: a prospective volumetric analysis. *Journal of Oral and Maxillofacial Surgery*.

[4] Arends C. R., Lindhout J. E., van der Molen L., Wilthagen E. A., van den Brekel M. W. M., Stuiver M. M. (2023). A systematic review of validated assessments methods for head and neck lymphedema. *European Archives of Oto-Rhino-Laryngology*.

[5] Koçer G., Sönmez S., Findik Y., Yazici T. (2020). Reliability of the linear measurement (contact) method compared with stereophotogrammetry (optical scanning) for the evaluation of edema after surgically assisted rapid maxillary expansion. *Healthcare*.

[6] Buitenhuis M. B., Klijn R. J., Rosenberg A. J. W. P., Speksnijder C. M. (2022). Reliability of 3D stereophotogrammetry for measuring postoperative facial swelling. *Journal of Clinical Medicine*.

[7] Ullah R., Turner P. J., Khambay B. S. (2015). Accuracy of three-dimensional soft tissue predictions in orthognathic surgery after Le Fort I advancement osteotomies. *The British Journal of Oral & Maxillofacial Surgery*.

[8] Böckmann R., Meyns J., Dik E., Kessler P. (2014). The modifications of the sagittal ramus split osteotomy: a literature review. *Plastic and Reconstructive Surgery–Global Open*.

[9] De Vet H. C. W., Terwee C. B., Mokkink L. B., Knol D. L. (2011). Validity. *Measurement in Medicine: A Practical Guide*.

[10] Hidding J. T., Viehoff P. B., Beurskens C. H. G., van Laarhoven H. W. M., Nijhuis-van der Sanden M. W. G., van der Wees P. J. (2016). Measurement properties of instruments for measuring of lymphedema: systematic review. *Physical Therapy*.

[11] Smith B. G., Lewin J. S. (2010). Lymphedema management in head and neck cancer. *Current Opinion in Otolaryngology & Head and Neck Surgery*.

[12] De Vet H. C. W., Terwee C. B., Mokkink L. B., Knol D. L. (2021). Responsiveness. *Measurement in Medicine: A Practical Guide*.

[13] Kau C. H., Cronin A., Durning P., Zhurov A. I., Sandham A., Richmond S. (2006). A new method for the 3D measurement of postoperative swelling following orthognathic surgery. *Orthodontics and Craniofacial Research*.

[14] Chiu C. S. W., Clark R. K. F. (1991). Reproducibility of natural head position. *Journal of Dentistry*.

[15] Chotipanich A., Kongpit N. (2020). Precision and reliability of tape measurements in the assessment of head and neck lymphedema. *PLoS One*.

[16] Beurskens A., de Vet H. C. W., Köke A. J. A. (1996). Responsiveness of functional status in low back pain: a comparison of different instruments. *Pain*.

[17] Kamper S. J., Ostelo R. W. J. G., Knol D. L., Maher C. G., de Vet H. C. W., Hancock M. J. (2010). Global perceived effect scales provided reliable assessments of health transition in people with musculoskeletal disorders, but ratings are strongly influenced by current status. *Journal of Clinical Epidemiology*.

[18] Hauke J., Kossowski T. (2011). Comparison of values of Pearson’s and Spearman’s correlation coefficients on the same sets of data. *Quaestiones Geographicae*.

[19] Taylor R. (1990). Interpretation of the correlation coefficient: a basic review. *Journal of Diagnostic Medical Sonography*.

[20] Goodwin L. D., Leech N. L. (2006). Understanding correlation: factors that affect the size of R. *The Journal of Experimental Education*.

[21] Wong J. Y., Oh A. K., Ohta E. (2008). Validity and reliability of craniofacial anthropometric measurement of 3D digital photogrammetric images. *The Cleft Palate-Craniofacial Journal*.

[22] Farah M. J., Wilson K. D., Drain M., Tanaka J. N. (1998). What is “special” about face perception?. *Psychological Review*.

[23] Vermaire J. A., Raaijmakers C. P. J., Verdonck-de Leeuw I. M. (2021). Mastication, Swallowing, and salivary flow in patients with head and neck cancer: objective tests versus patient-reported outcomes. *Supportive Care in Cancer*.

[24] Pedersen A., Wilson J., McColl E., Carding P., Patterson J. (2016). Swallowing outcome measures in head and neck cancer-how do they compare?. *Oral Oncology*.

[25] Hayhurst K. P., Massie J. A., Dunn G., Lewis S. W., Drake R. J. (2014). Validity of subjective versus objective quality of life assessment in people with schizophrenia. *BMC Psychiatry*.

[26] Speck R. M., Courneya K. S., Mâsse L. C., Duval S., Schmitz K. H. (2010). An update of controlled physical activity trials in cancer survivors: a systematic review and meta-analysis. *Journal of Cancer Survivorship*.

[27] White C. P., Hitchcock C. L., Vigna Y. M., Prior J. C. (2011). Fluid retention over the menstrual cycle: 1-year data from the prospective ovulation cohort. *Obstetrics and Gynecology International*.

[28] Bracher A., Knechtle B., Gnädinger M. (2012). Fluid intake and changes in limb volumes in male ultra-marathoners: does fluid overload lead to peripheral oedema?. *European Journal of Applied Physiology*.

